# Mechanisms of hemispheric specialization: Insights from analyses of connectivity

**DOI:** 10.1016/j.neuropsychologia.2006.07.002

**Published:** 2007

**Authors:** Klaas Enno Stephan, Gereon R. Fink, John C. Marshall

**Affiliations:** a Wellcome Department of Imaging Neuroscience, Institute of Neurology, University College London, 12 Queen Square, London WC1N 3BG, UK; b Institute of Medicine (IME), Research Centre Jülich, 52425 Jülich, Germany; c Department of Neurology, University of Cologne, 50937 Cologne, Germany; d Neuropsychology Unit, University Department of Clinical Neurology, Radcliffe Infirmary, OX2 6HE Oxford, UK

**Keywords:** Lateralization, Effective connectivity, Dynamic causal modeling, Corpus callosum, Inter-hemispheric integration

## Abstract

Traditionally, anatomical and physiological descriptions of hemispheric specialization have focused on hemispheric asymmetries of local brain structure or local functional properties, respectively. This article reviews the current state of an alternative approach that aims at unraveling the causes and functional principles of hemispheric specialization in terms of asymmetries in connectivity. Starting with an overview of the historical origins of the concept of lateralization, we briefly review recent evidence from anatomical and developmental studies that asymmetries in structural connectivity may be a critical factor shaping hemispheric specialization. These differences in anatomical connectivity, which are found both at the intra- and inter-regional level, are likely to form the structural substrate of different functional principles of information processing in the two hemispheres. The main goal of this article is to describe how these functional principles can be characterized using functional neuroimaging in combination with models of functional and effective connectivity. We discuss the methodology of established models of connectivity which are applicable to data from positron emission tomography and functional magnetic resonance imaging and review published studies that have applied these approaches to characterize asymmetries of connectivity during lateralized tasks. Adopting a model-based approach enables functional imaging to proceed from mere descriptions of asymmetric activation patterns to mechanistic accounts of how these asymmetries are caused.

## 1 Introduction

Traditional approaches to characterizing hemispheric specialization have relied on four main approaches: (i) neuropsychological investigation of patients with brain lesions (Damasio & Damasio, 1989) or iatrogenic splits of the corpus callosum (Gazzaniga, 2000), (ii) psychological assessment of hemispheric performance differences using tachistoscopic visual or dichotic auditory stimulus presentation techniques (Hugdahl, 1988; Miran & Miran, 1984; Nagae & Moscovitch, 2002), (iii) *post-mortem* investigations of human brains that focus on differences in microstructural properties (e.g. cytoarchitecture, myeloarchitecture) between homotopic regions of the two hemispheres (Amunts et al., 1999; Amunts, Jäncke, Mohlberg, Steinmetz, & Zilles, 2000; Jenner, Rosen, & Galaburda, 1999) and (iv) in vivo studies of both structural and functional asymmetries using a variety of techniques, e.g. magnetic resonance (MRI) morphometry, positron emission tomography (PET), functional MRI (fMRI), electroencephalography (EEG) and magnetoencephalography (MEG). All these approaches have been complementary and enormously helpful in delineating brain asymmetries. Tachistoscopic/dichotic investigations of healthy volunteers provide a behavioral characterization of lateralized cognitive processes, treating the brain as a black box, whereas neuropsychological, anatomical and physiological approaches describe hemispheric asymmetries in terms of *local* properties of the neurobiological “machinery”, i.e. regional asymmetries in functional involvement, cortical structure or neuronal activity, respectively. This article reviews the current state of an additional approach that is gaining increasing importance. This approach, the study of asymmetries of brain connectivity, goes beyond a mere description of hemispheric asymmetry and aims at clarifying its functional principles and computational mechanisms.

In this article, after discussing the historical origins of the concept of lateralization, we briefly review recent evidence from anatomical and developmental studies that hemispheric asymmetries in structural connectivity are a fundamental constraint of brain architecture and may be the cause for functional hemispheric specialization. These hemispheric differences in structural connectivity, which have been found both at the level of intra-areal microcircuits and inter-regional connections, are likely to form the structural substrate of different functional principles of information processing in the two hemispheres. We review how functional imaging data can be analyzed by models of functional and effective connectivity in order to characterize these functional principles. To familiarize the reader with the strengths, assumptions and limitations of available models of functional and effective connectivity, we briefly discuss several established methods, in particular structural equation models and dynamic causal models, which can be applied to data obtained from different functional imaging techniques. Finally, we review the results of several published studies that have successfully used models of functional and effective connectivity to address questions of hemispheric specialization and highlight how some of these models start to merge methodologically with other approaches from computational neuroscience. The focus is strictly on direct and quantitative measures of intra- and inter-hemispheric functional coupling derived from fMRI and PET data. In contrast, it is beyond the scope of this article to discuss the rich literature of analysis of functional coupling in terms of coherence or phase synchrony as measured by EEG or MEG (see Varela, Lachaux, Rodriguez, & Martinerie, 2001, for review). Also, we do not cover those approaches that are only indirectly related to analyses of connectivity, e.g. transcranial magnetic stimulation (TMS) or electroencephalographic latency differences.

Overall, we hope to convince the reader that formal system models, fitted to neuroimaging data of lateralized cognitive processes, are a useful, and indeed necessary, approach for lateralization research to proceed from mere descriptions of asymmetric activation patterns to mechanistic accounts of how these asymmetries are caused.

## 2 Historical origins of the concept of hemispheric specialization

The Hippocratic physicians should have discovered cerebral lateralization of language in the brain: they observed that injury to one side of the head was associated with contralesional hemiparesis. And that right hemiparesis was often associated with disturbance of speech. But they never linked the two phenomena, presumably because it seemed theoretically parsimonious that two very similar anatomical structures (the cerebral hemispheres) would have equally similar cognitive functions. The ventricular theory, expounded by Herophilus of Alexandria (circa 300 BCE), in which distinct psychological faculties such as imagination, conceptual thought and memory were represented in midline structures of the brain (the ventricles), also militated against any conception of left–right functional asymmetry (Marshall, 1977). Two millennia later, Thomas Willis (1621–1675) could see no virtue in the ventricular theory but continued to assign the seat of imagination to a midline structure: the corpus callosum. A century later, the French anatomist Felix Vicq d’Azyr argued that “the commissures are intended to establish sympathetic communications between different parts of the brain” (see Joynt, 1974). The corpus callosum connecting the right hemisphere with the left must, he argued, “play an important role in the unknown mechanism” of cerebral functions (Joynt, 1974). The crucial word here is clearly “unknown”.

One might have expected that Franz Joseph Gall (1757–1828) would conjecture, in the early nineteenth century, that different cognitive organs (modules) were punctately localised in different hemispheres. After all, Gall did describe a very perspicuous case of circumscribed amnestic aphasia after unilateral left frontal lesion. But he too was overly impressed by the apparent symmetry of the cerebral hemispheres. Thus, rather than give up his postulate that all mental organs were bilaterally represented, Gall argued that a sudden insult to one hemisphere would “upset the balance between the hemispheres, thus affecting the faculties on both sides” (Finger, 2000). This early concept of diaschisis is, of course, interesting and important in its own right. But the consequences of its deployment in this context meant that it would be Broca (1863) who convinced the neurological world that unilateral lesions of the left inferior frontal convolution (and not the right) gave rise to loss of “the memory of the procedure that is employed to articulate language” (Marshall & Fink, 2003).

Broca's paper opened the floodgates to the discovery of other lateralized impairments of higher mental functions (and, by extrapolation, lateralized cognitive modules representing those functions). There followed, for example, reports of spatial impairment after right posterior damage (Jackson, 1876), impairment of language comprehension after left temporal damage (Wernicke, 1874) and impairment of skilled praxis after left parietal damage (Liepmann, 1905). Studies of disconnection syndromes in which two relatively intact modules (that should interact) became isolated from each other due to commissural lesion concentrated for the most part on intra-hemispheric connectivity. The best known nineteenth century example was, of course, conduction aphasia consequent upon lesion of the arcuate fasciculus which disconnected Wernicke's area from Broca's area (Wernicke, 1874). There were, however, also some convincing examples of disorders that implicated inter-hemispheric commissures. Dejerine (1892) showed that alexia without agraphia could arise from the combination of lesions to the left occipital cortex and the splenium of the corpus callosum. The intact visual word form centre in left temporal–parietal cortex was thereby cut off from input from both the left and right visual fields. Likewise, Liepmann and Maas (1907) reported failure of the left hand to execute commands given verbally after callosal lesion. Psycho-physical evidence for the time course of normal callosal transmission was then obtained by Poffenberger (1912).

Despite the general agreement that the adult human brain is strongly characterized by hemispheric specialization, there has been comparatively little discussion of how or why relatively punctuate unilateral localisation of function should be found. Freud (1891) speculated that it would be sensible biological engineering to have Broca's area in close anatomical proximity to the motor strip representation of the vocal tract, and likewise sensible to have Wernicke's area adjacent to primary auditory cortex. Later, Lashley (1937) conjectured that “separate localisation of functions is determined by the existence of diverse kinds of integrative mechanisms which cannot function in the same field without interference”. Consistent with Lashley's argument, it is frequently claimed that fine motor control of the midline structure such as the vocal tract will be more effective if the command and control centre is unilaterally placed. Otherwise, conflict or noise could arise if two Broca's areas in opposite hemispheres were attempting to control speech production.

Although lateral specialization of function seems to be a fact, the execution of many even moderately complex tasks will draw upon some modules that are left-lateralized and some that are right-lateralized. As nineteenth and early twentieth century behavioral neurologists began to realize, this will require the transmission of structured information between the hemispheres (Liepmann, 1912; Poffenberger, 1912). In addition to this information transfer theory, two other concepts of hemispheric interactions have become important themes in laterality research: inter-hemispheric inhibition and hemispheric recruitment. These different concepts, all of which emphasize the relevance of connectivity for lateralization of brain function, will be discussed in more detail in the section on connectivity studies investigating inter-hemispheric integration below.

It is obvious from the examples of lateralized disconnection syndromes like conduction aphasia and from the importance of inter-hemispheric interactions that brain connectivity must play a fundamental role in hemispheric specialization. In particular, hemispheric specialization may be more appropriately characterized in terms of structural and functional connectional asymmetries between hemispheres rather than in terms of asymmetries in the local structure or intrinsic function of homotopic regions (Crow, 2005; McIntosh et al., 1994; Stephan et al., 2003; Stephan, Penny, Marshall, Fink, & Friston, 2005). With this notion gaining increasing importance in laterality research, the following sections of this article review the current state of efforts to (i) characterize hemispheric asymmetries in structural connectivity, within and between regions and (ii) to infer mechanistic principles of lateralization from functional neuroimaging and neurophysiological data using analyses of effective connectivity.

## 3 Asymmetries in structural brain connectivity

Structural asymmetries of the human brain have been described in various forms and at different scales. The comparison of homotopic regions in the two hemispheres has disclosed differences that range from dendritic tree features (Seldon, 1981), neuronal cell size (Hutsler & Gazzaniga, 1996) and cytoarchitecture (Amunts et al., 1999, 2000; Jenner et al., 1999) to differences in location, shape or volume of areas, sulci, gyri or whole lobes (see Toga & Thompson, 2003, for a comprehensive review). Several studies have found structural brain asymmetries, in terms of gyrification, regional volumes or white matter microstructure, to be expressed early during human ontogenesis (e.g. Chi, Dooling, & Gilles, 1977; de Lacoste, Horvath, & Woodward, 1991; Galaburda, LeMay, Kemper, & Geschwind, 1978; Gupta et al., 2005; Witelson & Pallie, 1973). The question is what developmental mechanisms underlie these asymmetries. This section discusses the currently available evidence that initial asymmetries in connectivity may, at least in part, cause other asymmetries of brain structure, both with regard to cytoarchitecture and macroscopic properties.

Our present understanding of brain development implies that these mechanisms are likely to consist of a mixture of intrinsic and extrinsic processes (Sur & Rubenstein, 2005). Intrinsic processes are those that induce regional parcellations within the cortical progenitor zone (the epithelium of the neural tube) whose neurons eventually migrate to form the cortex in an inside-out layered fashion. The existence of several molecules involved in the formation of such regional parcellations is well-established (Donoghue & Rakic, 1999; Fukuchi-Shimogori & Grove, 2001; Rubenstein et al., 1999). Processes that affect this parcellation of the progenitor zone differentially between hemispheres could lead to hemispheric differences in the microstructure or size of cortical regions. Indeed, a developmental study in rats provides some evidence that this type of process contributes to establishing microstructural asymmetries (Rosen, Sherman, & Galaburda, 1991). In contrast, extrinsic processes comprise changes due to the inputs conveyed by thalamo-cortical (and other) connections. Elegant experiments have shown that different cortices can radically change their microstructure and functional properties when they are surgically connected to different sensory inputs (Schlaggar & O’Leary, 1991). For example, primary auditory cortex develops the cytoarchitectonic and functional features of primary visual cortex, including functional orientation columns, when receiving retinal inputs after surgical rerouting in early development (Newton, Ellsworth, Miyakawa, Tonegawa, & Sur, 2004; Sur & Leamey, 2001). Similarly, many normal processes in cortical development depend on activity-dependent synaptic plasticity that induces strong microstructural changes, e.g. concerning the size and shape of dendritic trees (Cohen-Cory, 2002; Hua & Smith, 2004).

Overall, independently or additionally to regional parcellations within the cortical progenitor zone, structural hemispheric asymmetry between homotopic areas can result if the areas differ significantly in one or several of the three following factors: (i) their afferent connectivity, (ii) the sensory inputs conveyed by those connections or (iii) mechanisms of synaptic plasticity that translate these differences in inputs into microstructural changes. The role of the first factor, i.e. afferent connectivity per se, is highlighted by the studies cited above (Sur & Leamey, 2001). The importance of the third factor for microstructural features of cortex is emphasized by multiple studies that show changes in neuronal morphology, e.g. changes in dendritic tree size, after experimental manipulations of synaptic plasticity, e.g. blockage of NMDA receptors (Monfils & Teskey, 2004; Monfils, VandenBerg, Kleim, & Teskey, 2004). An intriguing demonstration of the second factor, i.e. the role of sensory inputs which are conveyed by connections and induce experience-dependent forms of synaptic plasticity, is provided by animal experiments in different species. For example, chicken and pigeon embryos are usually positioned such that only the right eye is exposed to light. This stimulates the growth of different visual projections systems in the left, as compared to the right, hemisphere and leads to pronounced functional differences in the visual performance of the hemispheres (Koshiba, Nakamura, Deng, & Rogers, 2003; Manns & Güntürkün, 1999; Rogers, 1990; Rogers & Deng, 1999). These brain asymmetries can be completely reversed, both structurally and functionally, if the normal lateralization of sensory inputs during development is altered (see Halpern, Güntürkün, Hopkins, & Rogers, 2005, for review). Another interesting phenomenon that is likely to result from experience-dependent plasticity is that musicians with absolute pitch have increased left–right asymmetries of planum temporale volume compared to musicians without absolute pitch or non-musicians (Schlaug, Jäncke, Huang, & Steinmetz, 1995; Keenan, Thangaraj, Halpern, & Schlaug, 2001).

Both mechanisms, regional differences in the progenitor zone and connectivity-dependent restructuring and plasticity, are now widely accepted as co-existing processes responsible for structural and functional patterning of the cortex during brain development (Redies, Treubert-Zimmermann, & Luo, 2003; Sur & Rubenstein, 2005). In addition to the studies cited above, the relevance of these approaches for the expression of brain asymmetry has been demonstrated by recent molecular developmental studies. For example, a comprehensive study of prenatal gene expression by Sun et al. (2005) have found a large number of genes that are asymmetrically expressed in corresponding parts of left and right human cortex at 12, 14 and 19 weeks after gestation, respectively. They focused on one particular gene, LMO4, which was differentially expressed in left and right perisylvian cortex at 12 and 14 weeks after gestation. The authors concluded that “the left–right differences in LMO4 expression in humans could potentially reflect either a differing topographic mapping in the two hemispheres or a difference in the tempo of cortical development …”. Given the importance of connectivity for both types of processes and the well-established role of LMO4 in neuritogenesis (Manetopoulos, Hansson, Karlsson, Jonsson, & Axelson, 2003; Vu et al., 2003), one may speculate that these hemispheric asymmetries in LMO4 expression contribute to differences in the development of connectivity in the two hemispheres. Of further interest is the additional finding of Sun et al. (2005) that *N*-cadherin and interacting molecules like CREB are also differentially expressed in left and right perisylvian cortex during development (see supplementary information to Sun et al., 2005). This is interesting because *N*-cadherin is crucially involved in both brain connectivity development and synaptic plasticity (Huntley, Gil, & Bozdagi, 2002; Salinas & Price, 2005; see also Garcia-Castro, Vielmetter, & Bronner-Fraser, 2000 who found that *N*-cadherin also regulates the asymmetry of visceral organs like the heart during development). Overall, even though there are currently only very few studies on the role of individual molecules in the development of brain asymmetry, the available data seem consistent with a critical role of connectivity for the development of hemispheric asymmetries.

Whatever the exact developmental mechanisms, the existence of hemispheric differences in structural connectivity, particularly but not exclusively with regard to language-relevant areas, have been clearly demonstrated, both in the fetal and the adult human brain. This finding has been made possible by two recent methodological advances that allow one to investigate structural connectivity in the human brain, albeit at very different levels of resolution: post-mortem delineation of microcircuits by means of lipophilic dyes (Galuske, Schlote, Bratzke, & Singer, 2000) and in vivo fiber tracking based on non-invasive diffusion weighted imaging (DWI; Behrens et al., 2003; Mori & Barker, 1999; Parker et al., 2002).

Galuske et al. (2000) used refined post-mortem tracing techniques to characterize the cortical microcircuitry in the language-relevant Wernicke region, the posterior part of the superior temporal gyrus and the posterior temporal plane, corresponding to the posterior part of Brodmann's area 22 (Brodmann, 1909). This area had previously been shown to possess microstructural (Hutsler & Gazzaniga, 1996) and macroscopic (Galaburda et al., 1978) hemispheric asymmetries, and its critical role for the auditory analysis of speech has been repeatedly demonstrated (Binder et al., 1997; Narain et al., 2003). Galuske et al. (2000) found a regularly spaced pattern of columnar neuronal clusters, corresponding to cortical macrocolumns, around their injection sites. While the cluster sizes did not differ between hemispheres, the average distance between these clusters was significantly larger in the left hemisphere. Due to their simultaneous use of multiple dyes, Galuske et al. (2000) could further demonstrate that the clusters were not all part of a single microcircuit but formed multiple independent (i.e. not directly connected) subsystems. They were thus able to conclude that the larger inter-column spacing in the left hemisphere would provide a structural basis for implementing a larger number of independent subsystems per volume unit. In analogy to visual cortex, where increases in inter-column spacing have been found in higher visual areas of the visual cortex (Amir, Harel, & Malach, 1993), it has been speculated that this higher number of subsystems, each of them specialized for processing particular features of the auditory input, could allow for representation of more complex auditory feature constellations (Galuske et al., 2000; Hutsler & Galuske, 2003). This increase in the range of computational complexity could explain the superiority of left Wernicke's area in the analysis of speech, compared to its right counterpart.

**Fig. 1:**
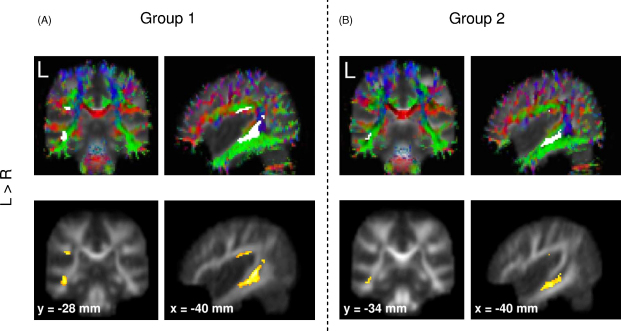
Results from a study of healthy adults by Büchel et al. (2004) who applied voxel-based morphometry to fractional anisotropy (FA), a diffusion-based measure of white matter microstructure (Pierpaoli & Basser, 1996). Testing for hemispheric differences in FA across the whole brain, they found a selective increase in the left arcuate fasciculus compared to the right (*p* < 0.05, whole-brain corrected for multiple comparisons). After initially demonstrating this asymmetry in a group of 15 volunteers (A), they subsequently replicated this finding in an independent group of 28 volunteers (B). Figure reproduced with permission from Oxford University Press.

The above findings at the level of cortical microcircuits have been complemented by several DWI studies. In addition to the study by Gupta et al. (2005), who demonstrated the presence of differences in frontal white matter microstructure in fetal brains, several DWI studies of the adult brain have shown hemispheric asymmetries in different properties of fiber tracts connecting posterior temporal and inferior frontal cortex, particularly the arcuate fasciculus (Fig. 1). Büchel et al. (2004) applied voxel-based morphometry (VBM) to whole-brain maps of fractional anisotropy (FA), a diffusion-based measure of white matter microstructure (Pierpaoli & Basser, 1996). Testing for hemispheric differences in white matter microstructure (and correcting for multiple comparisons) across the whole brain, they found a selective increase in FA in the left arcuate fasciculus compared to the right (see Fig. 1). Parker et al. (2005) used algorithmic tractography to trace connections between Wernicke's and Broca's regions in both hemispheres. They found two separate pathways, a dorsal one corresponding to the arcuate fasciculus and a ventral one that connects the two regions via the external capsule, uncinate fasciculus and the medial superior temporal gyrus. The ventral pathway was only found in the left hemisphere, and the connection strengths were overall higher in the left than in the right hemisphere. Nucifora, Verma, Melhem, Gur, and Gur (2005) performed a hypothesis-driven investigation of the arcuate fasciculus by means of diffusion tensor tractography. They demonstrated a higher fiber density in the left as compared to the right arcuate fasciculus.

Several DWI and post-mortem studies have also found asymmetries of fiber tracts between areas unrelated to language. For example, a post-mortem study of the human uncinate fasciculus, based on stereological methods, found a significantly larger volume and a significantly higher fiber density of the right as compared to the left uncinate fasciculus (Highley, Walker, Esiri, Crow, & Harrison, 2002). Another post-mortem study delineated the optic radiation histologically in 10 human brains and found that the volume of the left optic radiation was significantly higher than the right (Bürgel, Schormann, Schleicher, & Zilles, 1999). Gong et al. (2005) used DWI to investigate the symmetry of the cingulum, a prominent fiber tract near the midline of the brain, and found significantly higher FA for most parts of the left cingulum. A structure-function correlation approach was chosen by Tuch et al. (2005) who focused on the relation between microstructural properties of white matter tracts, as characterized by FA, and behavioral performance, measured in terms of reaction times (RTs), on a speeded visuospatial attention task. They found a significant correlation between individual RTs and FA values in fiber tracts involved in visuospatial attention, including the right optic radiation and white matter tracts located near right posterior thalamus and right medial precuneus WM. Although the lateralization of RT–FA correlations to right visual and parietal WM pathways is compatible with the specialization of right visual and parietal cortices for visuospatial attention, the unexpected aspect of their results was that the correlation was positive, i.e. higher FA was associated with longer RTs. While this appears to rule out a simple interpretation of FA as a microstructural measure primarily determined by the degree of myelinization, other potential explanations have been offered, e.g. a higher proportion of large caliber axons in the right visuospatial pathways which could allow for more diffusion orthogonal to the main direction of the axons (see Tuch et al., 2005, for details).

For completeness, it should finally be mentioned that despite the large literature on brain connectivity in non-human primates as assessed by invasive tract tracing studies, rather little attention has been devoted to asymmetries in primate brain connectivity. While the large majority of studies do not even indicate whether injections or labeled neurons were located in the left or right hemisphere, the few studies so far that have explicitly investigated connectional asymmetries have failed to find any (e.g. Cavada & Goldman-Rakic, 1989; McGuire, Bates, & Goldman-Rakic, 1991).

## 4 Characterizing the functional consequences of asymmetric structural connectivity

### 4.1 The need for formal system models

Altogether, the anatomical studies described in the previous section demonstrated hemispheric differences in the adult human brain, both in intra- and inter-areal connectivity and particularly with regard to areas involved in language. Given the dependency of information processing by neuronal units on their connectivity (Passingham, Stephan, & Kötter, 2002; Young, Hilgetag, & Scannell, 2000), these asymmetries in connectivity suggest differences in the computational principles used by the left and right hemisphere, particularly with regard to the processing of language-associated stimuli. What exactly these principles are, however, cannot be inferred from knowing anatomical connectivity alone, even if this knowledge was perfect. We also need to know the functional properties of the individual connections, e.g. whether they convey linear or non-linear effects, how strong these effects are and whether they happen almost instantaneously or with a delay. Systems with identical structural connectivity can show entirely different behavior if the functional properties of the connections are changed (Strogatz, 2001).

Therefore, if we want to understand the functional consequences of hemispheric asymmetries in structural connectivity we need to characterize the functional properties of connections in the system, for example, in terms of the synaptic strength of individual connections and how these change depending on the computational context (task requirements, learning, etc.). Connection strengths and other parameters (e.g. delay terms) can only be estimated from empirical observations of the neural system of interest. Therefore, testing specific hypotheses about the consequences of hemispheric differences in connectivity requires one to measure the system in action, e.g. using electrophysiological or functional imaging techniques, and explain mathematically how the observed system behavior is generated as a function of the structure of the system and the inputs it receives. Ideally, we therefore need formal system models in order to explain hemispheric differences in terms of functional principles (Stephan, 2004).

But what exactly is a “system” and why is the systems concept so useful for framing scientific questions? One could informally define a system as being a set of elements which interact with each other in some spatially and temporally specific fashion. More formally, a *system* can be defined as a set of elements with *n* time-variant properties that interact with each other. Each time-variant property *x*_*i*_ (1 ≤ *i* ≤ *n*) is called a *state variable*, and the *n*-vector *x*(*t*) of all state variables in the system is called the *state vector* (or simply *state*) of the system at time *t*:

(1) $$x(t)=\left[\begin{array}{c} x_{1}(t)\\ \vdots \\ x_{n}(t) \end{array}\right]$$

Taking an ensemble of interacting neurons as an example, the system elements would correspond to the individual neurons, each of which is represented by one or several state variables. These state variables could refer to various neurophysiological properties of the neurons, e.g. postsynaptic potentials, status of ion channels, etc. The crucial point is that the state variables interact with each other, i.e. the evolution of each state variable usually depends on other state variables. These functional dependencies between the state variables of the system have to be specified mathematically which requires a set of parameters *θ*. In neural systems, these parameters comprise at least the synaptic strengths of the connections between the system elements. Furthermore, we must not forget that biological systems are not autonomous but interact with their environment and that external perturbations have a considerable impact on the dynamics of the system. We, therefore, need to consider the input into the system, e.g. sensory information entering the brain. For a given system model, the set of all *m* known inputs can be represented by the *m*-vector function *u*(*t*). Assuming a deterministic behavior of the system, one can thus formulate a very general state equation for non-autonomous dynamic systems (see Stephan, 2004 for details and assumptions underlying the mathematical form chosen here):

(2) $$\frac{dx}{dt}=F(x,u,\theta )$$

Any model following this general schema provides a causal description of how system dynamics result from system structure, because it describes (i) when and where external inputs enter the system and (ii) how the state changes induced by these inputs evolve in time depending on the system's structure. As explained below in more detail, Eq. (2) therefore provides a general form for so-called models of *effective connectivity* in neural systems, i.e. the causal influences that neural units exert over another (Friston, 1994).

### 4.2 System concepts in functional neuroimaging

Modern cognitive neuroscience has adopted an explicit system perspective. A commonly accepted view is that the brain regions that constitute a given system are computationally specialized, but that the exact nature of their individual computations depends on context, e.g. the inputs from other regions. The aggregate behavior of the system depends on this *neural context*, the context-dependent interactions between the system components (McIntosh, 2000). An equivalent formulation of this perspective is provided by the twin concepts of functional specialization and functional integration (Friston, 2002b). *Functional specialization* assumes a local specialization for certain aspects of information processing but allows for the possibility that this specialization is anatomically segregated across different cortical areas. The majority of current functional neuroimaging experiments have adopted this view and interpret the areas that are jointly correlated to a certain task component as the elements of a distributed system which represents the neural basis of that task. However, this explanation is incomplete as long as no insight is provided into how the locally specialized computations are bound together by context-dependent interactions between these areas, i.e. the *functional integration* within the system.

The concepts of functional specialization and functional integration are highly relevant for questions on functional brain asymmetries. Conventional functional neuroimaging studies on hemispheric specialization have usually compared, explicitly or implicitly, the degree of functional specialization exhibited by homotopic regions, e.g. activation of left but not right Broca's area during semantic processing (Bookheimer, 2002; Hagoort, Hald, Bastiaansen, & Petersson, 2004) or predominant activation of the right as compared to left parietal areas during visuospatial attention tasks (Corbetta & Shulman, 2002; Fink et al., 2000). As already implied by the term “hemispheric specialization”, the notion of functional specialization can also be applied to a whole hemisphere, e.g. by classifying the response profiles of left and right hemisphere as “analytic” and “holistic”, respectively (Bradshaw & Nettleton, 1981).

Characterizing hemispheric asymmetries in terms of functional specialization alone, however, is insufficient. Functional integration is also fundamentally important for lateralized processes, both within a hemisphere (e.g. the functional cooperation of different language areas in the left hemisphere) and across hemispheres (e.g. the binding of processes lateralized to opposite hemispheres), and it is this aspect of hemispheric lateralization that we wish to highlight here. Generally, functional integration within distributed neural systems can be characterized in two ways, functional connectivity and effective connectivity (Friston, 1994). In the remainder of this article, we will first look in detail at some established approaches for characterizing functional and effective connectivity and then review studies which have applied these models to questions of functional integration during lateralized cognitive processes.

### 4.3 Analyses of functional connectivity

Functional connectivity is operationally defined as the temporal correlation between spatially segregated neurophysiological processes (Friston, 1994). For example, considering two voxels X and Y with time series {*x*_*t*_} and {*y*_*t*_}, the functional connectivity between the two voxels simply corresponds to the Pearson correlation coefficient *r* of the two time series

(3) $$r_{xy}=\frac{\text{co}{\text{v}}_{xy}}{s_{x}\cdot s_{y}}$$

where *s*_*x*_ and *s*_*y*_ are the standard deviations and cov_*xy*_ is the covariance of the two time series. Note that functional connectivity suffers from the general problem of interpreting correlations: are the two time series correlated because (i) X influences Y, (ii) Y influences X, (iii) both influence each other or (iv) both are functionally unrelated but similarly influenced by a third variable? Disambiguating these options requires a model of the causal influences, i.e. effective connectivity (see below).

One approach to applying the concept of functional connectivity to PET and fMRI data is to choose a particular reference voxel and compute, for the whole brain, the correlation of all other voxel time series with this seed voxel time series (Bokde, Tagamets, Friedman, & Horwitz, 2001; Horwitz, Rumsey, & Donohue, 1998). An alternative is to characterize orthogonal components of the temporal covariance matrix by decomposing it into its eigenvectors, e.g. using singular value decomposition (Friston, 1994). A related approach is partial least squares (McIntosh & Lobaugh, 2004), a technique which has found multiple applications in the analysis of neuroimaging data.

### 4.4 Models of effective connectivity

In contrast to functional connectivity, the notion of effective connectivity is based on a model of the causal influences between the elements of a system (Friston, 1994). Therefore, there is no single mathematical definition for effective connectivity; instead, a variety of different models of effective connectivity have been proposed (for overviews, see Friston, 2002b; Stephan, 2004). Models of effective connectivity describe the mechanisms that determine the dynamics of neural systems, i.e. how activity induced by external inputs is propagated within the system according to its connectivity. It is therefore useful to consider each particular implementation of effective connectivity as a special case of Eq. (2) (see Stephan, 2004, for a more detailed exposition), and this is the perspective we will take here. In this section, we briefly summarize three commonly used models of effective connectivity which were used by the studies discussed in the following sections of this paper: psycho-physiological interactions (PPI), structural equation modeling (SEM) and dynamic causal modeling (DCM).

#### 4.4.1 Psycho-physiological interactions

For the regression-like model used by PPI the static form of Eq. (2) is appropriate, i.e. *x*(*t*) = *F*(*x*, *u*, *θ*, *t*) which assumes that the system is at equilibrium at each point of observation (see appendix to Friston, Harrison, & Penny, 2003). Introduced by Friston et al. (1997), PPI are one of the simplest models available to assess functional interactions in neuroimaging data. Given a chosen reference time series *y*_0_ (obtained from a reference voxel or region), PPI computes whole-brain connectivity maps of this reference voxel with all other voxels *y*_*i*_ in the brain according to the regression-like equation

(4) $$y_{i}=ay_{0}+b(y_{0}\times u)+cu+X\beta +\varepsilon$$

Here, *a* is the strength of the intrinsic (context-independent) connectivity between *y*_0_ and *y*_*i*_. The bilinear term *y*_0_ × *u* represents the interaction between physiological activity *y*_0_ and a psychological variable *u* which can be construed as a contextual input into the system, modulating the connectivity between *y*_0_ and *y*_*i*_ (× represents the Hadamard product, i.e. element-by-element multiplication). The third term describes the strength *c* by which the input *u* evokes activity in *y*_*i*_ directly, independent of *y*_0_. Finally, *β* are parameters for effects of no interest *X* (e.g. confounds) and *ɛ* is an error term. Although this is a very simple and non-dynamic model, PPI do contain the basic components of system descriptions as outlined above (see Eq. (2)). There is also a general similarity between the form of Eq. (4) and that of the state equation of DCM (Eq. (7), see below). However, since only pair-wise interactions between the reference voxel and all other brain voxels are considered, this model is rather restricted in its ability to represent real neural systems. Although PPIs are therefore not a full system model, they have a very useful role in exploring the context-dependent functional interactions of a chosen region across the whole brain. This exploratory nature bears some similarity to analyses of functional connectivity. Unlike analyses of functional connectivity, however, PPIs represent the contextual modulation of connectivity, and this modulation has a directional character, i.e. testing for a PPI from *y*_0_ to *y*_*i*_ is not identical to testing for a PPI from *y*_*i*_ to *y*_0_. This is because regressing *y*_0_ × *u* on *y*_*i*_ is not equivalent to regressing *y*_*i*_ × *u* on *y*_0_.

#### 4.4.2 Structural equation modeling

After having been used in the social sciences for several decades, SEM was introduced to neuroimaging in the early 1990s by McIntosh and Gonzalez-Lima (1991). It is a multivariate, hypothesis-driven technique that is based on a structural model which represents the hypothesis about the causal relations between several variables (see Büchel & Friston, 1997; Bullmore et al., 2000; McIntosh & Gonzalez-Lima, 1994; Penny, Stephan, Mechelli, & Friston, 2004a, for methodological details). In the context of neuroimaging, these variables are the measured time series *y*_1_, …, *y*_*n*_ of *n* brain regions and the hypothetical causal relations are based on anatomically plausible connections between the regions. The strength of each connection *y*_*i*_ → *y*_*j*_ is specified by a so-called “path coefficient” which, similarly to a partial regression coefficient, indicates how the variance of *y*_*j*_ depends on the variance of *y*_*i*_ if all other influences on *y*_*j*_ are held constant.

One way to summarize the statistical model of SEM implementations for neuroimaging data is given by the equation

(5) y=Ay+u

where *y* is the *n* × *s* matrix of *n* area-specific time series with *s* scans each, *A* the *n* × *n* matrix of path coefficients (with zeros for non-existent connections) and *u* is the *n* × *s* matrix of “innovations”, i.e. zero mean Gaussian error terms, which are driving the modeled system (Penny et al., 2004a; see also McIntosh & Gonzalez-Lima, 1994, for an equivalent formulation). Parameter estimation rests on minimizing the difference between the observed and the predicted covariance matrix *Σ* of the areas (Bollen, 1989). *Σ* can be computed by transforming Eq. (5):

(6) $$y={\left(I-A\right)}^{-1}u;\;\Sigma =yy^{\text{T}}={\left(I-A\right)}^{-1}uu^{\text{T}}{\left(I-A\right)}^{-1^{\text{T}}}$$

where *I* is the identity matrix and ‘T’ denotes the transpose operator. The first line of Eq. (6) can be understood as a generative model of how system function results from the system's connectional structure: the measured time series *y* results by applying a function of the inter-regional connectivity matrix, i.e. (*I* − *A*)^−1^, to the Gaussian innovations *u*.

It is beyond the scope of this paper to discuss SEM in full methodological detail and the reader is referred to the large body of existing literature (e.g. Bollen, 1989; McIntosh & Gonzalez-Lima, 1994; Penny et al., 2004a). One particular detail, however, that is important for studies of hemispheric specialization is the limitation of SEM to models of relatively low complexity. The problem is that models with reciprocal connections and loops easily become non-identifiable (see Bollen, 1989, for details). Given that callosal connections seem to be generally reciprocal and one usually needs to study bidirectional interactions between the hemispheres, this constraint is particularly problematic for models of inter-hemispheric integration. Heuristics for dealing with complex models have been established that use multiple fitting steps in which different parameters are held constant while changing others (see McIntosh et al., 1994, for an example), yet this constraint has been a limiting factor for the application of SEM to questions on inter-hemispheric integration.

#### 4.4.3 Dynamic causal modeling

An important limitation of the models discussed so far is that they operate at the level of the measured signals. Taking the example of fMRI, the model parameters are fitted to BOLD series which result from a haemodynamic convolution of the underlying neural activity. Any inference about inter-regional connectivity obtained by PPI or SEM is only an indirect one because these models do not include the forward model linking neuronal activity to the measured haemodynamic data. The causal architecture of the system that we would like to identify, however, is expressed at the level of neuronal dynamics. Therefore, to enable inferences about connectivity between neural units we need models that combine two things: (i) a parsimonious but neurobiologically plausible model of neural population dynamics and (ii) a biophysically plausible forward model that describes the transformation from neural activity to the measured signal. Such models make it possible to fit jointly the parameters of the neural and of the forward model such that the predicted time series are optimally similar to the observed time series. In principle, any of the models described above could be combined with a modality-specific forward model. So far, however, dynamic causal modeling is the only approach where the marriage between models of neural dynamics and biophysical forward models is a mandatory component. DCM has been implemented both for fMRI (Friston et al., 2003) and EEG/MEG data (David et al., 2006). For simplicity, we here only briefly summarize the implementation of DCM for fMRI.

DCM for fMRI offers a simple model for the neural dynamics in a system of *n* interacting brain regions. It models the change of a neural state vector *x* in time, with each region in the system being represented by a single state variable, using the following bilinear differential equation:

(7) $$\frac{dx}{dt}=F(x,u,\theta^{n})=\left(A+\sum_{j=1}^{m}u_{j}B^{\left(j\right)}\right)x+Cu$$

Note that this neural state equation follows exactly the general form for deterministic system models introduced by Eq. (2). Here, the neural state variables represent a summary index of neural population dynamics in the respective regions. The neural dynamics are driven by experimentally controlled external inputs that can enter the model in two different ways: they can elicit responses through direct influences on specific regions (e.g. evoked responses in early sensory cortices; the *C* matrix) or they can modulate the coupling among regions (e.g. during learning or attention; the *B* matrices). The neural parameters *θ*^*n*^ = {*A*, *B*, *C*} can be expressed as partial derivatives of *F* (*n* in *θ*^*n*^ is not an exponent but a superscript that denotes “neural”):

(8) $${\left(A=\frac{\partial F}{\partial x}\right|}_{u=0};\;B^{\left(j\right)}=\frac{\partial^{2}F}{\partial x\partial u_{j}};\;C={\left(\frac{\partial F}{\partial u}\right|}_{x=0}$$

The matrix *A* represents the effective connectivity among the regions in the absence of input, the matrices *B*^(*j*)^ encode the change in effective connectivity induced by the *j*th input *u*_*j*_ and *C* embodies the strength of direct influences of inputs on neuronal activity.

DCM for fMRI combines this model of neural dynamics with an experimentally validated haemodynamic model that describes the transformation of neuronal activity into a BOLD response. This “Balloon model” was initially formulated by Buxton, Wong, and Frank (1998) and later extended by Friston, Mechelli, Turner, and Price (2000). Briefly, it consists of a set of differential equations that describe the relations between four haemodynamic state variables, using five parameters (*θ*^*h*^). Changes in neural activity elicit a vasodilatory signal that leads to increases in blood flow and subsequently to changes in blood volume and deoxyhemoglobin content. The predicted BOLD signal is a non-linear function of blood volume and deoxyhemoglobin content. Details of the haemodynamic model can be found in other publications (Friston et al., 2000; Stephan, Harrison, Penny, & Friston, 2004).

The neural and haemodynamic parameters *θ* = {*θ*^*n*^, *θ*^*h*^} are jointly estimated from the measured BOLD data, using a fully Bayesian approach with empirical priors for the haemodynamic parameters and conservative shrinkage priors for the coupling parameters. Details of the parameter estimation scheme, which rests on a gradient ascent procedure embedded into an expectation maximization (EM) algorithm and uses a Laplace (i.e. Gaussian) approximation to the true posterior, can be found in Friston (2002a). Eventually, the posterior distributions of the parameter estimates can be used to test hypotheses about connection strengths. Usually, these hypotheses concern context-dependent changes in coupling. If there is uncertainty about the connectional structure of the modeled system, or if one would like to compare competing hypotheses (represented by different DCMs), a Bayesian model selection procedure can be used to find the DCM that exhibits an optimal balance between model fit and model complexity (Penny, Stephan, Mechelli, & Friston, 2004b).

### 4.5 Neurobiological interpretability of models of effective connectivity

One may wonder what degree of neurobiological interpretability the models of effective connectivity discussed above possess. DCM is particularly relevant in this discussion because it is currently the only model of effective connectivity for fMRI data that explicitly models the neural level. DCM for fMRI is obviously not specified at a level of neurobiological finesse that allows one to distinguish between different processes at synaptic, cellular, columnar or laminar levels. Instead, the mechanisms represented by DCM, e.g. context-dependent changes of particular connection strengths, refer to the level of large neural populations contained by one or several voxels (note that even a single standard size voxel contains hundreds of thousands of neurons). However, this relatively high degree of abstraction present does not mean that the causal mechanisms modeled by DCM are neurobiologically meaningless. Many of the processes that one typically models with DCM, e.g. changes in synaptic strength during learning or context-specific modulation of connections due to attention or other cognitive factors, have been investigated at the level of single neurons or microcircuits by invasive recording experiments (e.g. Luck, Chelazzi, Hillyard, & Desimone, 1997), and DCMs provide a simple mechanistic description of these processes at the level of neuronal populations. In particular, the distinction between direct and modulatory effects in DCMs represents a direct analogy at the population level to the concept of driving and modulatory afferents in studies of single neurons (Sherman & Guillery, 1998). A more detailed discussion of these issues can be found elsewhere (Penny et al., 2004b; Stephan, 2004). Finally, one should mention that much more fine-grained DCMs have been developed than for fMRI, for example, for EEG/MEG data. Here, each region is characterized by eight state variables that represent quite detailed components of the neurobiological machinery, including firing rates and membrane potentials of different neuronal units, e.g. pyramidal cell populations and inhibitory interneuron populations (David et al., 2006).

## 5 Functional imaging studies of brain connectivity in lateralized cognitive functions

### 5.1 Asymmetries of intra-hemispheric connectivity

Traditionally, as explained above, hemispheric specialization has been characterized in terms of asymmetries in the local structure or function of homotopic regions. An alternative approach that has gained momentum over the last years is the notion that lateralization may be more appropriately characterized in terms of connectivity asymmetries between hemispheres. In this section, we review some of the most influential neuroimaging studies of this kind which have used analyses of functional or effective connectivity. We restrict this review to those studies that explicitly assess asymmetries of intra-hemispheric connectivity. This excludes conventional activation studies in which co-activation of areas is interpreted as putative evidence for connectivity between them; this kind of analysis does not allow for unambiguous inference about connectivity (see Stephan, 2004, for a discussion of this point). Also, there are multiple elegant studies of effective connectivity during lateralized tasks that have deliberately restricted their connectivity analysis to the dominant hemisphere (e.g. Bitan et al., 2005; Coull, Büchel, Friston, & Frith, 1999; Mechelli, Penny, Price, Gitelman, & Friston, 2002; Smith, Stephan, Rugg, & Dolan, 2006); these studies are not discussed in detail either.

**Fig. 2:**
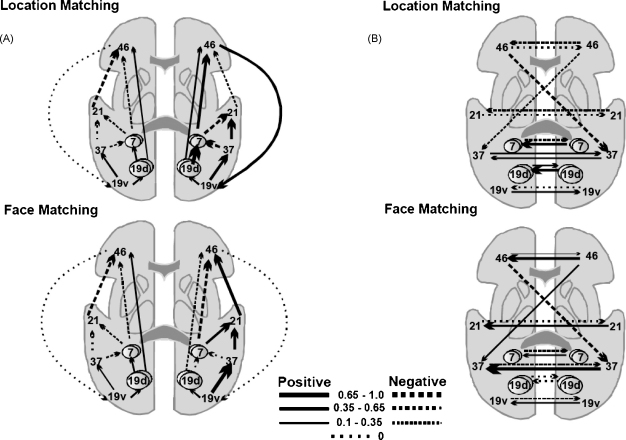
Results from structural equation modeling, applied to PET data from a study by McIntosh et al. (1994) who used two right-lateralized matching tasks for faces and locations. (A) The analysis of intra-hemispheric connectivity showed that the selective functional dependencies between ventral stream areas during the face matching task and between dorsal stream areas during the location matching task, respectively, were much stronger in the right than in the left hemisphere. (B) Strong asymmetries of the inter-hemispheric connection strengths, with right-to-left callosal connections between homotopic regions being positive and much stronger during both tasks than left-to-right connections. See main text for more details. Figure reproduced with permission from Springer Verlag.

A pioneering study of hemispheric differences in connectivity was conducted by McIntosh et al. (1994) who applied PET to two matching tasks for faces and locations where the volunteers had to choose which of two stimuli corresponded to a reference stimulus. Both face and location matching tasks are known to have a right-hemispheric dominance and should show a relative preference for engaging the ventral and dorsal stream of the visual system, respectively. Surprisingly, the activation pattern was found to be fairly bilateral for both tasks. A connectivity analysis using SEM revealed, however, that the selective functional dependencies between ventral stream areas during the face matching task and between dorsal stream areas during the location matching task, respectively, were much stronger in the right than in the left hemisphere (Fig. 2A). In fact, in the left hemisphere the two tasks did not differ with regard to the effective connectivity between visual areas. Furthermore, McIntosh et al. (1994) demonstrated top–down effects during the location matching task that were restricted to the right hemisphere, i.e. an influence of the right middle frontal gyrus (area 46) onto right extrastriate areas (Fig. 2A). This fronto-occipital top–down influence may represent the mechanism by which the right hemisphere alters early visual processing in accord with task demands.

**Fig. 3:**
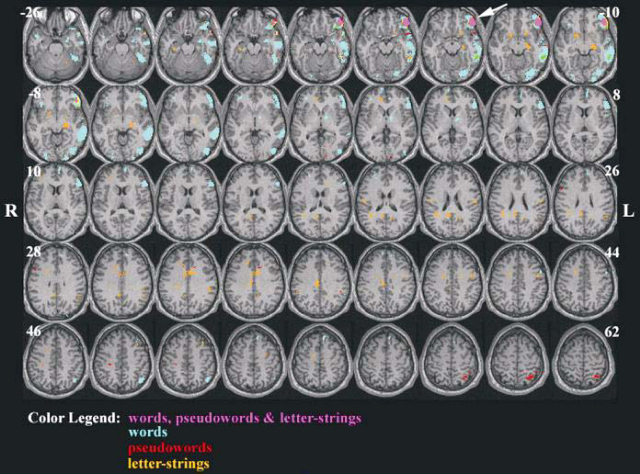
Functional connectivity of the left anterior inferior frontal gyrus (aIFG) during an orthographic task (Bokde et al., 2001). All voxels are highlighted in color whose BOLD signal correlated strongly positively (*r* > 0.4) with the BOLD signal in a reference voxel in left aIFG (white arrow). The color legend indicates for which type of stimuli (words, pseudowords and letter strings) these correlations were found. The image is shown according to radiological convention. Figure is reproduced from Bokde et al. (2001) with permission by Elsevier Ltd.

Complementary findings exist for tasks with left-hemispheric dominance, e.g. language paradigms. Horwitz and colleagues have demonstrated that even simple approaches to characterizing connectivity, i.e. seed voxel functional connectivity analyses, can contribute to a better understanding of lateralization during language processing. For example, Horwitz et al. (1998) used PET to compare dyslexic to healthy subjects during different reading tasks. In healthy subjects, they found the expected robust functional connectivity between the left angular gyrus and other reading-related areas in inferior frontal and temporal cortices. In dyslexic subjects, the left angular gyrus appeared to be disconnected from these areas. This functional disconnection in dyslexic patients is paralleled by a fractional anisotropy decrease in the same region, corresponding to a diminished microstructural integrity of white matter, which was found in a DWI study of adults with poor reading skills (Klingberg et al., 2000). Another study by Bokde et al. (2001) applied the same approach to fMRI data in a one-back orthographic matching task on different word stimuli (i.e. words, pseudowords, letter strings and false fonts; Tagamets, Novick, Chalmers, & Friedman, 2000). Bokde et al. (2001) investigated the hypothesis that the left anterior inferior frontal gyrus (aIFG) is involved in the semantic analysis of words whereas the left posterior inferior frontal gyrus (pIFG) plays a role in the phonological analysis of words. They found that left pIFG exhibited a pronounced functional connectivity with left temporal language areas during the presentation of all stimuli that could be processed phonologically (i.e. words, pseudowords, letter strings, but not false fonts). In contrast, left aIFG showed significant functional connectivity with these areas only during the presentation of real words, but not during processing of pseudowords, letter strings and false fonts, none of which have a semantic content (see Fig. 3). The critical point was that in both cases this pattern of functional connectivity was entirely restricted to the left hemisphere: analyses of the functional connectivity of the homotopic voxels in right aIFG and pIFG did not show any significant coupling with language-relevant temporal areas. Altogether, studies of the kind described above demonstrate how hemispheric specialization can be conceptualized in terms of hemispheric differences in the functional integration of cooperating areas.

Beyond language, attention plays a particular role for the discussion of connectivity and lateralization. Selective attention provides one of the best studied examples of context-dependent changes in connection strength (e.g. Büchel & Friston, 1997; Friston et al., 2003), and some recent studies have begun to characterize how selective attention in visual and auditory space is associated with a corresponding connection strengths increase in the associated hemisphere. For example, a PET study of dichotic listening employed a PPI analysis to show that selective orientation towards one ear as (compared to a control condition with identical words presented to both ears, evoking a centrally located fused percept) led to increases in the effective connectivity of the superior temporal gyrus and the intra-parietal sulcus with other regions, but only within the hemisphere contralateral to the attended ear (Lipschutz, Kolinsky, Damhaut, Wikler, & Goldman, 2002). An elegant fMRI study by Haynes, Tregellas, and Rees (2005) used four rotating spirals, one in each of the four quadrants of the visual fields. During central fixation, the volunteers were instructed to attend covertly to two of the four spirals at a time and decide whether their directions of rotation were identical or opposite. Using DCM, they found that the change in spatial attention was paralleled by a change in the connection strength between the retinotopically corresponding parts of areas V1 and V2. For example, when subjects covertly compared the two spirals in the left visual field, the functional coupling increased between the corresponding retinotopically mapped parts of V1 and V2 in the right hemisphere (and vice versa for attention to the spirals in the right visual field). Corresponding effects were found when subjects attended to two spirals in opposite hemifields; then, the inter-hemispheric coupling between the retinotopic representations increased.

Although spatial attention can induce changes in connectivity in both hemispheres as described above, there is good evidence that several right-hemispheric areas, particularly frontal eye field (FEF), intra-parietal sulcus and temporo-parietal junction (TPJ), play a dominant role in the actual implementation of spatial attention, regardless where it is directed (Corbetta & Shulman, 2002; Fink et al., 2000; Fink, Marshall, Weiss, & Zilles, 2001; Gitelman et al., 1999; Halligan, Fink, Marshall, & Vallar, 2003; Marshall & Fink, 2001). These right-hemispheric areas are thus likely candidate sources of the modulatory effects exerted by spatial attention on connection strengths throughout the brain. Yet, other than the study by McIntosh et al. (1994) described above, there is surprisingly limited work so far that provides direct evidence for this notion. An fMRI study by Gitelman, Parrish, Friston, and Mesulam (2002) that examined changes in connectivity of the superior colliculus between overt visuospatial search and a saccade control condition by means of PPI gave mixed results, showing similar degrees of collicular coupling with left- and right-hemispheric areas, with the exception that only the right, but not left, FEF increased its coupling with the superior colliculus during overt visuospatial search relative to controlled saccades. Another study by Ruff and Driver (2006) investigated whether anticipation of a distractor stimulus, located in the opposite hemifield to the target stimulus, would alter the spatiotopic activations elicited by anticipation of the target. Using fMRI, they demonstrated that both anticipation of targets and distractors induced activations in contralateral occipital cortex but there was no additional modulation of target anticipation by knowledge about the presence of a distractor. However, an analysis of functional coupling using PPI showed that the right, but not left, TPJ showed stronger functional coupling with occipital regions contralateral to the target, in both the left and the right hemisphere, during preparation for trials with an isolated target than for trials with an anticipated distractor. This pattern of connectivity is compatible with the putative role of right TPJ in bottom–up (stimulus-driven) rather than top–down attentional selection (Corbetta & Shulman, 2002), because in the paradigm by Ruff and Driver (2006) stimulus-driven direction of attention is likely to operate successfully only in the trials where no distractor is present.

**Fig. 4:**
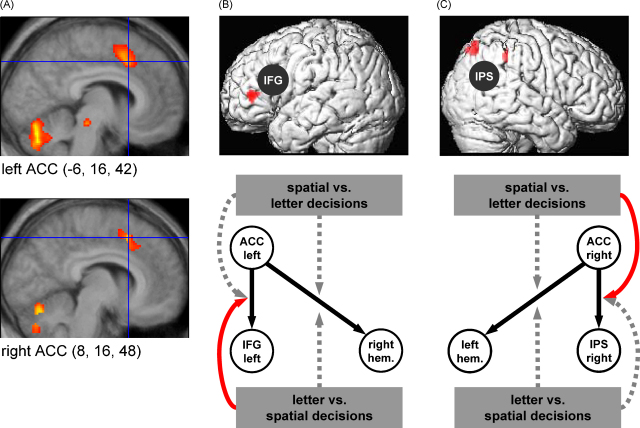
Schematic summary of a study by Stephan et al. (2003) that applied letter and spatial decision tasks to identical word stimuli. (A) Brain areas that were significantly activated during both letter and spatial decisions (contrast between the letter decision task and the baseline condition, masked by the contrast between the spatial decision task and the baseline condition; *p* < 0.05 whole-brain cluster-level corrected). Crosshairs highlight the anterior cingulate cortex (ACC) which was bilaterally activated in both conditions. (B) Results from a PPI analysis of the effective connectivity of the left ACC. Left ACC specifically increased its coupling with left inferior frontal gyrus during letter decisions (*p* < 0.05, small-volume corrected). Dashed arrows denote non-significant modulation of couplings. (C) Results from a PPI analysis of the effective connectivity of the right ACC. Right ACC specifically increased its coupling with anterior and posterior parts of right intra-parietal sulcus during spatial decisions (*p* < 0.05, small-volume corrected). Figure is adapted from Stephan et al. (2003), with permission by Science (The American Association for the Advancement of Science).

We conclude this section on asymmetries in intra-hemispheric connectivity by summarizing a recent fMRI study by Stephan et al. (2003). This study differs from the ones described above in that it simultaneously investigated lateralization of language and visuospatial processes. It addressed the unresolved question whether lateralization of brain activity inevitably depends on the nature of the sensory stimuli or can be determined solely by the nature of the cognitive task undertaken. For example, microstructural differences between hemispheres that favor the processing of certain stimulus characteristics and disadvantage others (Jenner et al., 1999) have been postulated to mediate stimulus-dependent lateralization in a bottom–up fashion (Sergent, 1983). In contrast, the processing demands of the task, mediated through cognitive control processes, might determine in a top–down fashion which hemisphere takes precedence over the other in accomplishing a given task (Fink et al., 1996; Levy & Trevarthen, 1976). To decide between these two competing views, Stephan et al. (2003) developed a paradigm in which the same type of stimuli was used throughout the experiment while different task instructions made subjects attend to certain stimulus features and ignore others. The stimuli consisted of concrete German nouns (each word composed of four letters) in which either the second or third letter was red. In a letter decision task, the subjects had to ignore the position of the red letter and indicated by button press whether or not the word contained the target letter “A”. In a visuospatial decision task, they were required to ignore the language-related properties of the word and to decide whether the red letter was located left or right of the word centre. The results of a conventional analysis, using statistical parametric mapping, were clearly in favor of the top–down hypothesis: despite the use of identical word stimuli in all conditions, comparing letter to visuospatial decisions showed strongly left-lateralized activity, including classical language areas like Broca's area in the left IFG, whereas comparing visuospatial to letter decisions showed strongly right-lateralized activity in the parietal cortex. What remained unclear from this analysis, however, were the mechanisms by which information processing was biased towards one hemisphere in a task-dependent fashion. The stimuli contained both letter and visuospatial information and thus required subjects to favor processing of that information which was meaningful for the current task and inhibit processing of the other information. Previous split-brain patient studies by Levy and Trevarthen (1976) had indicated that such a cognitive control process, i.e. the task-dependent simultaneous enhancement and inhibition of processing different stimulus features, might be the decisive “switch” that controlled the relative involvement of the two hemispheres. If this was true, this mechanism should lead to task- and hemisphere-specific changes in functional coupling between control areas in the frontal lobe and areas related to the execution of the tasks. In the study by Stephan et al. (2003), comparisons between the two tasks and a control condition (a simple reaction time task on the same type of stimuli) showed that the only putative control area was the anterior cingulate cortex (ACC). This area showed increased activity in *both* hemispheres during *both* tasks (Fig. 4A). However, when ACC connectivity with the rest of the brain was analyzed by means of PPIs (Friston et al., 1997), a striking hemispheric dissociation was found: left ACC specifically increased its coupling during letter decisions with the left IFG (Fig. 4B), an important language area, whereas the right ACC specifically increased its connectivity during visuospatial decisions with areas in the right parietal cortex known to be involved in spatial judgments (Fig. 4C). No other brain area showed significant task-dependent changes in coupling with either left or right ACC. As highlighted in a commentary to this study, these findings suggest that “processing is directed to either the left or the right hemisphere, depending on what needs to be done with the information” (McIntosh and Lobaugh, 2003). The critical aspect is that this mechanism of cognitive control could not be inferred from conventional activation maps; instead, how hemispheric asymmetry was controlled was only obvious in terms of the task-specific connectivity of the ACC.

### 5.2 Asymmetries of inter-hemispheric connectivity

One of the fundamental constraints of human brain function is the requirement to integrate processes from both hemispheres. Efficient inter-hemispheric integration would still be required even if the hemispheres were perfectly functionally symmetrical. This is illustrated by the simple example where one has to respond with a right arm movement (executed by the left motor cortex) to a visual stimulus presented in the periphery of the left visual (and thus received by the right visual cortex). Regardless of how symmetric the brain is, this situation requires stimulus information to be transferred from the right to the left hemisphere. It is likely that this need for inter-hemispheric integration is considerably amplified in an asymmetrically organized brain because it will often be the case that a cognitive operation will depend on subprocesses that are lateralized to opposite hemispheres. The above example brings about many fundamental questions, for example: when, how and where in the brain is information transferred between hemispheres? What determines whether the brain processes information in the hemisphere specialized for that information alone or whether it draws on additional computational resources in the less specialized hemisphere? How are simultaneous workings of the two hemispheres synchronized, e.g. to prevent interference of processes (see Lashley, 1937)?

These issues have been the subject of much theoretical work, and as a result three major complementary theories have been formulated that have guided investigations of inter-hemispheric integration. As mentioned in the historical section, the oldest concept is probably that of *information transfer* between the hemispheres (e.g. Poffenberger, 1912). In the context of lateralized tasks with hemisphere-specific inputs (e.g. peripheral visual presentation), this theory predicts that transfer of sensory information should be asymmetrically enhanced from the non-dominant to the dominant hemisphere to ensure maximally efficient processing in the specialized hemisphere (e.g. Endrass, Mohr, & Rockstroh, 2002; Nowicka, Grabowska, & Fersten, 1996). In terms of effective connectivity, it predicts a task-dependent increase in connectivity from the non-dominant to the dominant hemisphere but only when stimulus information is initially restricted to the non-dominant hemisphere.

A more recent and very influential concept has been the notion of *inter-hemispheric inhibition* (Kinsbourne, 1970). It has been agued that the regulatory mechanisms that “coordinate, select and integrate the processes subserved by each hemisphere” will also require a range of inter-hemispheric inhibitory mechanisms “to achieve unified performance from a bilateral system capable of producing simultaneous and potentially conflicting outputs” (Chiarello & Maxfield, 1996). This paper by Chiarello and Maxfield is an excellent review of the evidence for inter-hemispheric suppression, inter-hemispheric isolation and inter-hemispheric interference, with interesting suggestions about the functional significance of these mechanisms. With regard to connectivity, inter-hemispheric inhibition predicts a task-dependent symmetric pattern of negative connection strengths between hemispheres (strictly speaking, this requires the assumption that neural inhibition leads to a decrease in the measured signal).

The third major concept of inter-hemispheric integration concerns *hemispheric recruitment* or *processing mode setting*, i.e. whether information processing is restricted to a single hemisphere or distributed across both hemispheres. Behavioral evidence indicates that this is largely determined by the complexity (cognitive demand) of the task performed (Banich, 1998; Hellige, 1990). Several studies have shown that if the neural resources in the hemisphere receiving a stimulus are insufficient for optimal processing, the benefits of distributing the processing load across both hemispheres are likely to outweigh the costs of transcallosal information transfer (see Banich, 1998, for review). Given a sufficiently demanding task, this recruitment of an additional hemisphere even occurs during lateralized tasks when the dominant hemisphere receives the stimulus (Belger & Banich, 1998). This additional recruitment of the non-dominant hemisphere requires tight cooperation, i.e. functional coupling, of both hemispheres, regardless of which hemisphere initially received the stimulus. Two components are likely to be expressed in terms of task-dependent changes in effective connectivity: an increase of connections from the dominant to the non-dominant hemisphere that reflects the “recruitment” of the non-dominant hemisphere, and an increase of connection strengths in the opposite direction, induced by the non-dominant hemisphere “returning” the results of the computations delegated to it by the dominant hemisphere. Altogether, this cooperation is expected to be expressed either in terms of a symmetric task-dependent increase of connection strength between homotopic areas, or, if “recruitment” and “return” processes are spatially segregated, in asymmetric task-dependent increase of connection strength between different areas.

In the past, questions on inter-hemispheric interactions have been mainly addressed by means of elegant behavioral studies (for reviews, see Banich, 1998; Hellige, 1990; Liederman, 1998), studies of patients with callosal lesions (Corballis, Corballis, & Fabri, 2003; Funnell, Corballis, & Gazzaniga, 2000; Gazzaniga, 2000), EEG/MEG studies of inter-hemispheric coherence and synchrony (e.g. Schack, Weiss, & Rappelsberger, 2003) and invasive recording studies in animals (Cardoso de Oliveira, Gribova, Donchin, Bergman, & Vaadia, 2001; Engel, König, Kreiter, & Singer, 1991). All these methods have limitations. Behavioral studies cannot elucidate which neural processes generate the observed responses and where in the brain these processes happen. Callosal lesions are usually quite extended (particularly iatrogenic ones) and the considerable plasticity of the human brain complicates the interpretation of behavioral deficits in these patients. EEG/MEG studies suffer from the inverse problem, i.e. without strong a priori constraints, it is difficult to localize the sources that generate the measured responses. Although advanced methods for solving this problem are now available (e.g. Mattout et al., 2006), the large majority of available EEG/MEG studies on inter-hemispheric integration have analyzed functional coupling at the level of the sensor data only and hence do not allow for localization of the neural units that exhibit this coupling. Finally, invasive recordings are not possible in humans (with the exception of presurgical evaluation of epilepsy patients) and can only probe very few locations at a time.

While functional imaging techniques, particularly fMRI, overcome many of these issues and provide both whole-brain investigation and excellent spatial resolution, they are not free of problems when used to investigate inter-hemispheric integration by means of analyses of connectivity. A particular problem is that, due to the reciprocal nature of callosal connections and the multiple pathways by which two hemispheres can interact, models of inter-hemispheric integration are usually quite complex. PPI is too simple a model to allow for a satisfactory investigation of such systems. Also, SEM is only of limited help for complex models because of problems of identifiability, the simplest example being when there are more free parameters than empirically measured covariances (for discussions of this issue, see McIntosh & Gonzalez-Lima, 1994 and Penny et al., 2004a). Suggestions how to apply SEM to models of inter-hemispheric integration models have been made, e.g. to use an iterative fitting procedure in which intra-hemispheric parameters are estimated in a first pass and then kept fixed when extending the model to include inter-hemispheric connections (McIntosh et al., 1994) or to constrain the callosal connections to have the same path coefficient in both directions (Rowe et al., 2002; Schlösser et al., 2003). The latter approach, however, shares the problem with analyses of functional connectivity that asymmetries in inter-hemispheric influences cannot be investigated.

Focusing on studies that have specifically investigated such asymmetries in inter-hemispheric interactions, there are, to the best of our knowledge, at present only two studies in the literature that fulfill this criterion (McIntosh et al., 1994; Stephan et al., 2005). However, it can be expected that with the advent of DCM, which can deal with complex models, this number will substantially increase in the near future. Given the huge gap of our understanding about the functional principles of inter-hemispheric integration and how they relate to asymmetries of brain function, we hope that the discussion in this paper will contribute to stimulating future studies.

As already described in the section on intra-hemispheric connectivity, McIntosh et al. (1994) performed a PET study of face and location matching tasks. Although it is well-established that both tasks have a right-hemispheric dominance, the activation pattern was surprisingly bilateral for both tasks. A SEM connectivity analysis, however, not only showed higher functional coupling within the right as compared to the left hemisphere (see above), but also helped to understand the bilateral activation pattern from the initial conventional analysis. In their model, McIntosh et al. found strong asymmetries of the inter-hemispheric connection strengths, with right-to-left callosal connections between homotopic regions being positive and much stronger during both tasks than left-to-right connections (Fig. 2B). They concluded that the observed bilateral activation during the two right-lateralized tasks was due to a transcallosal recruitment of the non-dominant left hemisphere by the dominant right hemisphere. They could distinguish this interpretation from the alternative of simple information transfer because in their paradigm the stimuli were presented centrally and thus information was available to both hemispheres.

**Fig. 5:**
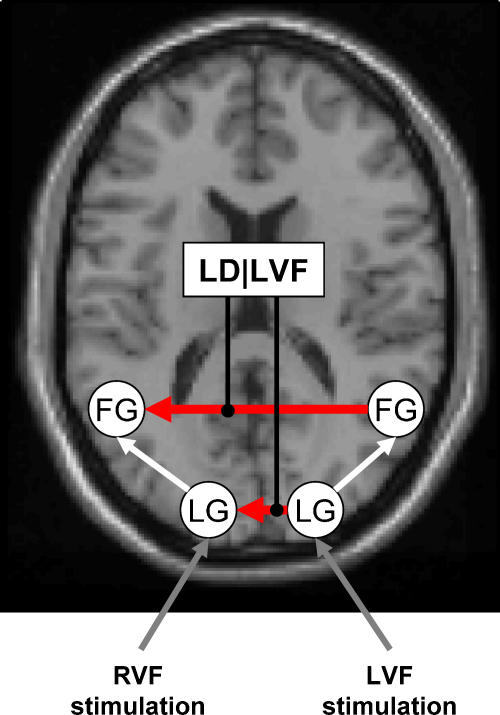
Schematic summary of the results from a study of hemispheric integration (Stephan et al., 2005) which applied DCM to data of a single subject from the group studied by Stephan et al. (2003; see Fig. 4). In this particular subject, inter-hemispheric connections between lingual gyri (LG) and fusiform gyri (FG), respectively, were modulated by letter decisions (LD), but conditional on stimuli being presented in the left visual field (LVF). This modulation was asymmetric, i.e. significantly stronger for right-to-left connections than vice versa (see Stephan et al., 2005, for details). This result, a task-dependent increase in connectivity from the non-dominant to the dominant hemisphere but only when stimulus information is initially provided to the non-dominant hemisphere, is in accordance with the theory that the corpus callosum subserves information transfer to the specialized hemisphere (see main text).

Stephan et al. (2005) presented the results from a DCM analysis of inter-hemispheric integration in the ventral stream of the visual system in a single subject, taken from the group described in Stephan et al. (2003). As described in the previous section, in this paradigm subjects had to make letter decisions or spatial decisions about identical word stimuli displayed in the peripheral visual fields. One of the aims of this experiment which could not be addressed by the initial SPM and PPI analyses reported by Stephan et al. (2003) was to determine (i) whether task or stimulus properties (i.e. visual field of presentation) determined the strength of callosal connections and (ii) which of the three theories of inter-hemispheric integration gave predictions that best fitted the observed inter-hemispheric dynamics between areas in the ventral stream of the visual system during letter decisions. To address these questions, Stephan et al. (2005) adopted the following strategy. First, they systematically constructed 16 competing models of inter-hemispheric interactions that covered all possible options concerning how task demands and visual field of stimulus presentation could affect functional coupling within and between hemispheres. After separately fitting all models to the same data, they used a Bayesian model selection procedure (Penny et al., 2004b) to determine which model exhibited the highest evidence, i.e. the probability of the data given by the model, and thus an optimal balance between model fit and model complexity. The final step consisted of statistical inference about the relevant model parameters, i.e. the modulation of callosal connections by task and/or visual field. Stephan et al. found that the best model was one in which callosal connections depended on the letter decision (LD) task, but conditional on the visual field of presentation: the connection from right to left lingual gyrus (LG) was strongly enhanced during the letter decision task but only when the stimuli were presented in the left visual field (LVF) and thus initially received by the right hemisphere (Fig. 5). In contrast, there was no significant modulation of the left-to-right LG connections. A posterior density analysis of the modulatory parameters confirmed the presence of this asymmetry with 98.7% probability. This result, a task-dependent increase in connectivity from the non-dominant to the dominant hemisphere but only when stimulus information is initially provided to the non-dominant hemisphere, perfectly fits the predictions from the information transfer theory outlined above. In the particular subject studied by Stephan et al. (2005), a similar, albeit weaker, asymmetry was observed with regard to the callosal connections at the level of the fusiform gyrus (FG; Fig. 5).

## 6 Investigating developmental changes in structural connectivity and their relation to functional lateralization

Collectively, the studies described in the sections above demonstrate that asymmetries of intra- and inter-hemispheric effective connectivity are a highly informative index of hemispheric specialization and go beyond the traditional approach of defining lateralization through asymmetries in the local structure or function of homotopic regions. As described in the section on asymmetries of structural brain connectivity and their developmental determinants above, it seems likely that asymmetries in effective connectivity can be causally related to asymmetries in structural connectivity forming during neurodevelopment. While animal studies strongly imply such a relation, definite proof for such a relation, for example, from longitudinal within-subject studies demonstrating a tight relation between developing asymmetries in structural and effective connectivity, has yet to be obtained for the human brain. This is due to methodological and ethical problems associated with longitudinal studies of human brain development. For obvious ethical reasons, any experimental procedure that is invasive (e.g. histological investigations to assess microstructural changes in connectivity) or that may indirectly affect development (e.g. pharmacological manipulations of molecular processes putatively involved in asymmetric formation of connections) are prohibited in humans. Unfortunately, the available non-invasive imaging procedures do not yet provide sufficient resolution that we could detect subtle changes in structural brain connectivity during the early stages of human brain development when decisive processes underlying lateralization presumably take place (see Gupta et al., 2005, for preliminary attempts to track prenatal connectivity changes using DWI). This is because high-resolution DWI data require high magnetic field strengths and/or long acquisition times, both of which are not permissible or practically feasible for perinatal imaging. Until better non-invasive methods are available for assessing structural connectivity with high resolution, studies of the relation between the development of structural brain asymmetries and the resulting changes of effective connectivity will have to focus on childhood and adolescence. In this period significant changes are still likely to occur, albeit probably at a slower rate than perinatally. Here, we describe two examples of possible research strategies.

First, it would be important to combine analyses of effective connectivity with DWI and morphometric studies that probe changes in hemispheric differences in gray and/or white matter properties over time. So far, to our knowledge, there is a complete lack of such studies. Morphometric measures such as cortical thickness may be particularly useful because there are widespread hemispheric differences (Luders et al., 2006), and a previous study indicated that inter-regional correlations in cortical thickness may be a function of inter-regional structural connectivity (Lerch et al., 2006). The latter could be tested directly in longitudinal studies that jointly investigate changes in cortical thickness, white matter organization and effective connectivity during childhood and adolescence. It would be informative to evaluate the results from such studies in reference to probabilistic cytoarchitectonic atlases, but so far these have only been developed for the adult human brain (cf. Eickhoff et al., 2005).

A second strategy rests on developmental studies of animals with different genetic status, e.g. knock-out models with regard to candidate genes (like LMO4 or *N*-cadherin, see above) for development of asymmetric brain connectivity. By testing for concomitant changes in structural connectivity (derived from quantitative tract tracing studies) and effective connectivity (estimated from neurophysiological measures) that follow experimental manipulations of experience-dependent plasticity, such studies could establish a direct role of current candidate genes for both the formation of asymmetric structural connectivity and for the subsequent functional expression of this asymmetry in terms of effective connectivity. Any positive findings could then be taken back to human studies which investigate, using functional imaging and genotyping, whether there is a statistical relationship between measures of effective connectivity during lateralized tasks and particular genetic haplotypes implicated by the animal models.

## 7 Summary and outlook

This review focused on the role of connectivity for understanding hemispheric specialization. We have reviewed evidence from recent anatomical and developmental studies that asymmetries in structural connectivity may be a key component in the development of hemispheric specialization. Such differences in anatomical connectivity, which have been described both within and between cortical areas, may represent the structural substrate of different styles of information processing in the two hemispheres. After a brief methodological overview of some commonly used models of connectivity, we reviewed published PET and fMRI studies that have applied these approaches to characterize asymmetries of intra- or inter-hemispheric connectivity during lateralized tasks. We hope that these examples have demonstrated three main things: first, that hemispheric specialization can be usefully defined by asymmetries of intra-hemispheric functional and effective connectivity and moreover that connectivity can be a more sensitive marker of hemispheric specialization than asymmetries of activation patterns (cf. McIntosh et al., 1994). Second, that analyses of connectivity can provide a mechanistic understanding of how lateralization can (sometimes) be entirely task-driven (cf. Stephan et al., 2003). And third, how models of effective connectivity can be used to infer functional principles of inter-hemispheric integration from neuroimaging data.

At the present time, many questions on hemispheric specialization are still open. For example, what are the exact computational advantages (and disadvantages) of an asymmetric brain? Is hemispheric specialization a developmental process that, once a certain stage has been reached, remains in a fixed state or is it a dynamic process? What role do synaptic plasticity and modulatory transmitter systems play? It will be important to address these questions, not only for our general understanding of human brain function, but also with regard to the many clinical disorders that implicate hemispheric asymmetries, either due to asymmetric lesions (like aphasia, apraxia or neglect) or due to an as yet unknown reason, as in dyslexia (Heim and Keil, 2006), autism (Herbert et al., 2005) and schizophrenia (Mitchell & Crow, 2005; Petty, 1999). For example, mechanistic models of hemispheric specialization may provide endophenotypes for better diagnosis and classification of diseases with diffuse diagnostic criteria like schizophrenia or autism (cf. Stephan, 2004). Moreover, good models may enable us to reverse-engineer asymmetric neural systems and teach us how to induce compensatory changes in case of disorders. With such models, it may be possible to derive better diagnostic tools for presurgical evaluation (Klöppel & Büchel, 2005), novel forms of rehabilitation training for brain-lesioned patients and predict advantageous consequences of physical (e.g. transcranial magnetic stimulation) or pharmacological manipulations. Whatever the exact research strategy chosen to pursue such goals, it seems likely that a computational systems perspective and a model-based approach will be necessary to enable neuroscience to proceed from mere descriptions of brain asymmetries to mechanistic accounts of how these asymmetries are caused and how they can be influenced.
